# Annual patterns of macroalgal blooms in the Yellow Sea during 2007–2017

**DOI:** 10.1371/journal.pone.0210460

**Published:** 2019-01-14

**Authors:** Jianheng Zhang, Jinting Shi, Song Gao, Yuanzi Huo, Jianjun Cui, Hui Shen, Guiyan Liu, Peimin He

**Affiliations:** 1 College of Marine Ecology and Environment, Shanghai Ocean University, Shanghai, China; 2 North China Sea Marine Forecasting Center of State Oceanic Administrator, Qingdao, China; 3 Jiangsu Institute Marine Fisheries, Nantong, China; National Taiwan Ocean University, TAIWAN

## Abstract

The world’s largest macroalgal blooms caused by *Ulva prolifera* have occurred in the Yellow Sea for 11 consecutive years. The area covered by blooms has been approximately 500 km^2^ in previous years, while in 2017, the maximum area decreased significantly to 312 km^2^. In this study, we concluded that species competition between *Ulva* and *Sargassum* (fast rise of the golden tides), extreme high sea surface temperature and harvest for floating *Ulva* macroalgae were the three critical factors influencing the sharp reduction in covered area for blooms in 2017. In addition, analysis of annual variations of *Pyropia* aquaculture area in the Southern Yellow Sea over the past two decades revealed that a great expansion in “Sansha” regions was mainly responsible for the initial blooms in 2007, and that this expansion supported the great biomass of the blooms in following years. Based on these findings, we suggest comprehensive utilization of the macroalgal blooms is a feasible way to control them.

## Introduction

Green tides, which are formed by rapid growth and vast accumulation of unattached green macroalgae, are associated with eutrophicated marine environments [1, 2]. Over the past few decades, green tides have been increasing in severity, frequency, and geographic range, resulting in their becoming a growing concern worldwide [3–6]. The world's largest green tide events, which are caused by *Ulva prolifera*, have occurred annually from 2007 to 2017 along the coast of the Yellow Sea, China, seriously affecting marine environments and ecological services functions [7, 8].

Green tides in the Yellow Sea are characterized by seasonal and annual occurrence, long distance transportation and large biomass. Over the past decade, domestic and overseas investigators have made thoroughly investigated this issue, and obtained many worthwhile results, resulting in the sources of green algae [9–14] and blooming mechanisms [15–20] being described in great detail.

Although the work reported in previous studies has led to a much better understanding of green tides in the Yellow Sea, many questions have yet to be answered. *Pyropia* aquaculture along the coast of the Southern Yellow Sea started in the 1970s and fouling green algae have always existed on the *Pyropia* aquaculture infrastructure, but no report of green tides were made until 2007. Thus, it is still not clear why the green tide started after 2007, although the rapid expansion, especially the great increase in *Pyropia* aquaculture areas in 2008, may be one of the responsible factors [21]. It is also not known why the area of green tides decreased by almost half in 2017 relative to previous years. One of the goals of green tides research is determining how to control it. Therefore, if the reason(s) for this significant reduction in green tides in 2017 could be identified, it may lead to development of methods for their elimination. Accordingly, in this study, we collected data and conducted field investigations to answer the two aforementioned questions.

## Materials and methods

The individual in this manuscript has given written informed consent (as outlined in PLOS consent form) to publish these case details.

### Maximum area of blooms as determined by satellite remote sensing

To determine the time series location of the maximum coverage area of the macroalgal blooms, we used satellite remote sensing methods. Daily HJ-1A/1B satellites images were obtained for the Yellow Sea in summer from 2008 to 2017. HJ-1A/1B satellites, which are the new generation of small Chinese civilian earth-observing optical remote sensing satellites, have wide-coverage multispectral charge-coupled device (CCD) cameras with a nadir pixel resolution of 30 m and central-pixel matching accuracy of 0.3 pixels. Images from these satellites were examined to search for days that were sufficiently cloud free to observe floating patches of algae if they existed at the time. The area covered with blooms was then measured and used to determine the maximum annual biomass.

### Total *Pyropia* aquaculture area determined by satellite remote sensing

The tidal flats along the Southern Yellow Sea are unique in terms of their huge geometric scale, abundant sediment supply associated with large rivers, silt-dominated sediments and offshore radial sand ridge [22]. Because of the complex sediment transport dynamics driven by the wave and current interactions, the tidal flats in this area are well-developed and range in width from several kilometers to tens of kilometers, which provides an ideal location for *Pyropia* aquaculture.

To estimate the total aquaculture area in recent years, multi-source satellite images including radar satellite ERS-2 PRI, Landsat TM/ETM, CBERS-2B and HJ-1A/1B, images were collected by the East China Sea Branch of the State Oceanic Administration of China. The obtained satellite images were then examined to identify days that were sufficiently cloud-free and to observe the rafts at low tide during *Pyropia* cultivation.

### Biomass measurement of attached *Ulva* in *Pyropia* aquaculture area

Semi-floating raft cultivation techniques have been widely adopted in these *Pyropia* farms, combining the strong points of the pillar and the floating methods, especially for intertidal cultivation. At high tide, nets float on the water, maximizing the light available to the seaweed, while at low tide the nets rest on the ground by short legs.

**Fig 1:**
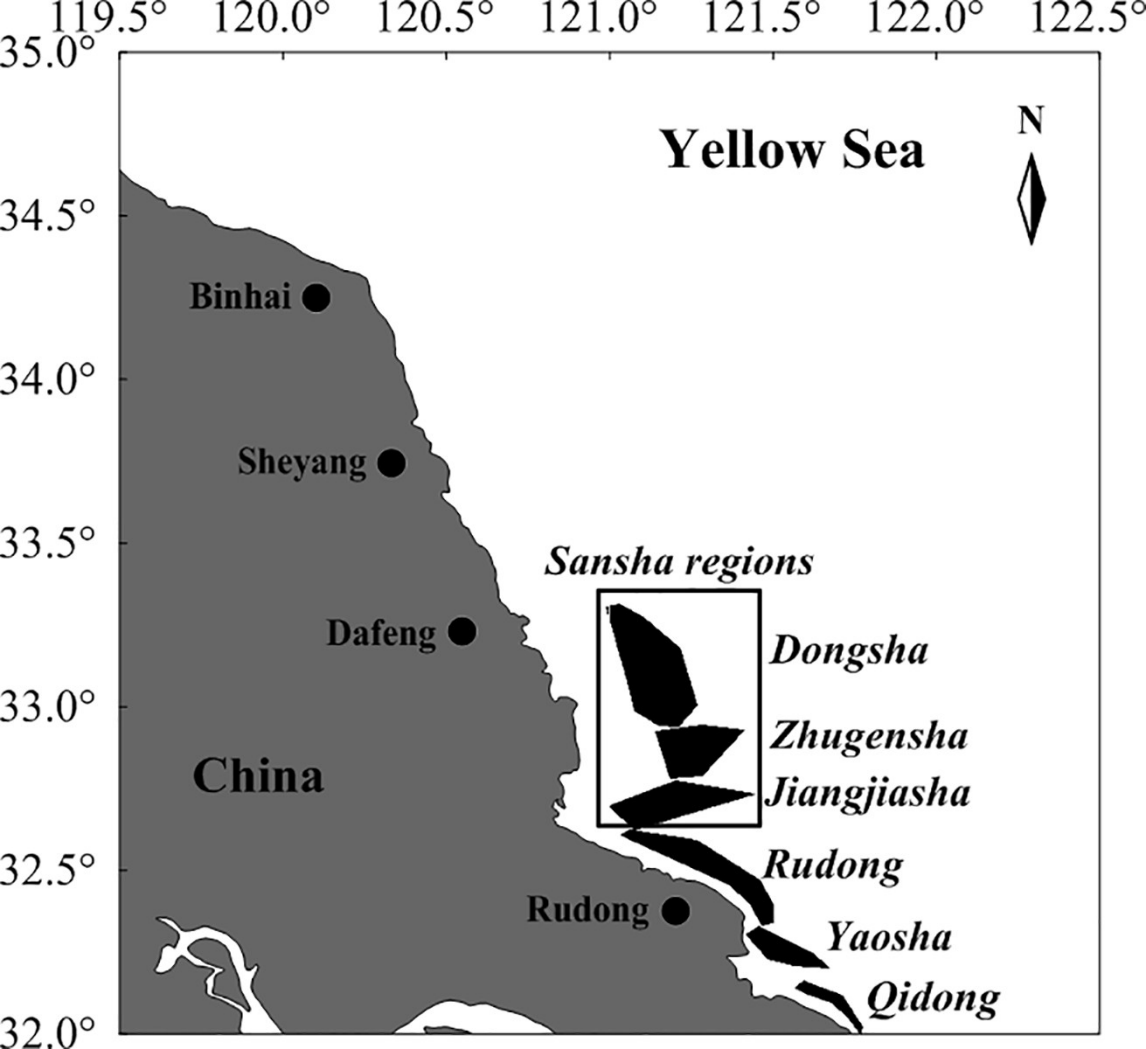
Map of *Pyropia* aquaculture area in the radial sand ridge area.

The entire aquaculture area was divided into six regions, Dongsha, Jiangjiasha, Zhugensha, Rudong, Yaosha and Qidong (Fig 1). In each region, ten rafts were randomly selected to collect samples of the attached green algae in early April 2013, 2014, 2016 and 2017. Each raft covered 7.5 m^2^ and consisted of two bamboo poles, two pieces of rope and one nursery net. All green algae on the selected rafts were removed to estimate the biomass of *Ulva* spp. and the total *Pyropia* aquaculture area was obtained from satellite remote sensing. These data were then used to calculate the total biomass of attached *Ulva* annually.

### Monthly average sea surface temperature (SST)

Monthly averaged night-time SST data for June and July from 2008 to 2017, the fast expansion season for the *Ulva* bloom, were obtained from the NASA data distribution system (http://oceancolor.gsfc.nasa.gov).

## Results

### Comparison of the maximum coverage area of the blooms from 2008 to 2017

**Fig 2:**
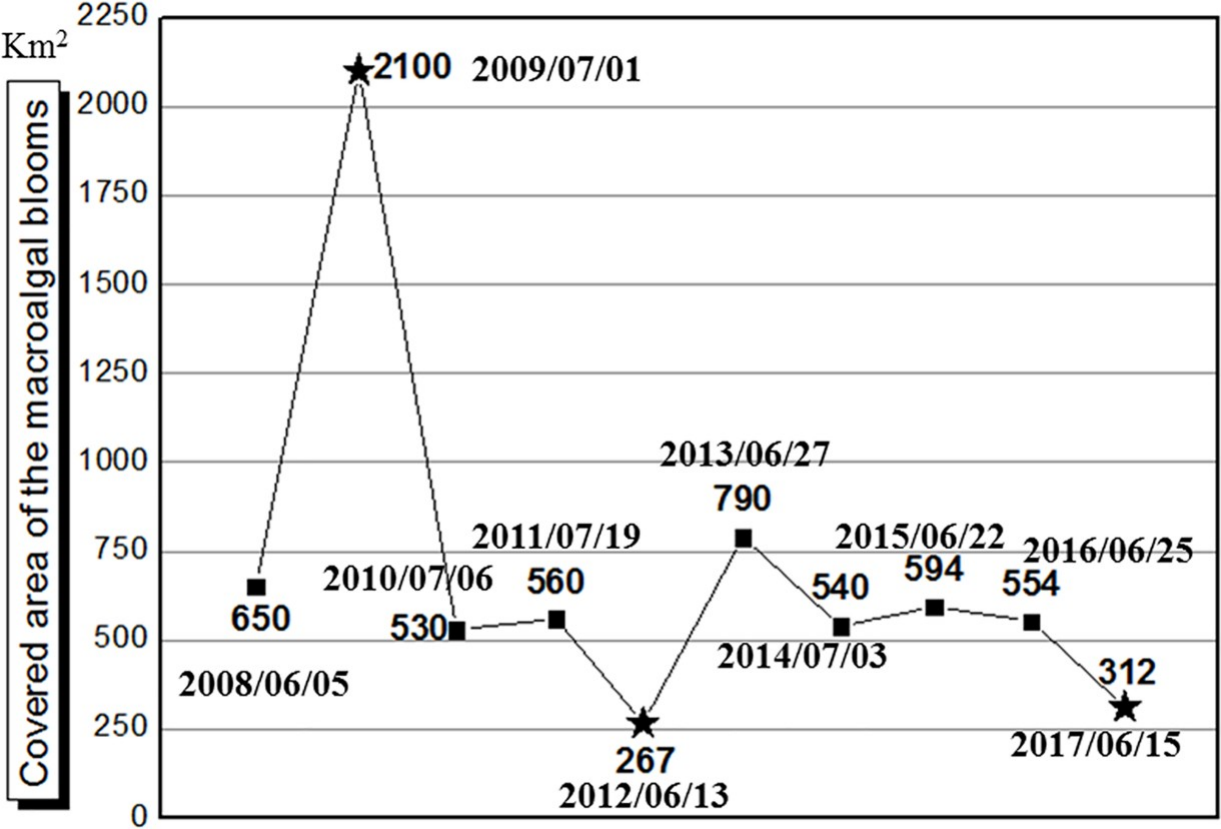
Variations in maximum covered area for macroalgal blooms during 2008–2017.

The maximum coverage areas of the floating blooms were 650 km^2^, 2100 km^2^, 530 km^2^, 560 km^2^, 267 km^2^, 790 km^2^, 540 km^2^, 594 km^2^, 554 km^2^ and 312 km^2^, respectively, which were all observed in the Northern Yellow Sea in June and July of each year (Fig 2). In comparison, it should be noted that the impact of the 2009 bloom was far more widespread than that of other years, while during 2012 and 2017 the amounts of floating *Ulva* biomass were reduced by almost half when compared to other years.

### Annual patterns of monthly averaged SST in June and July between 2008 and 2017

**Fig 3:**
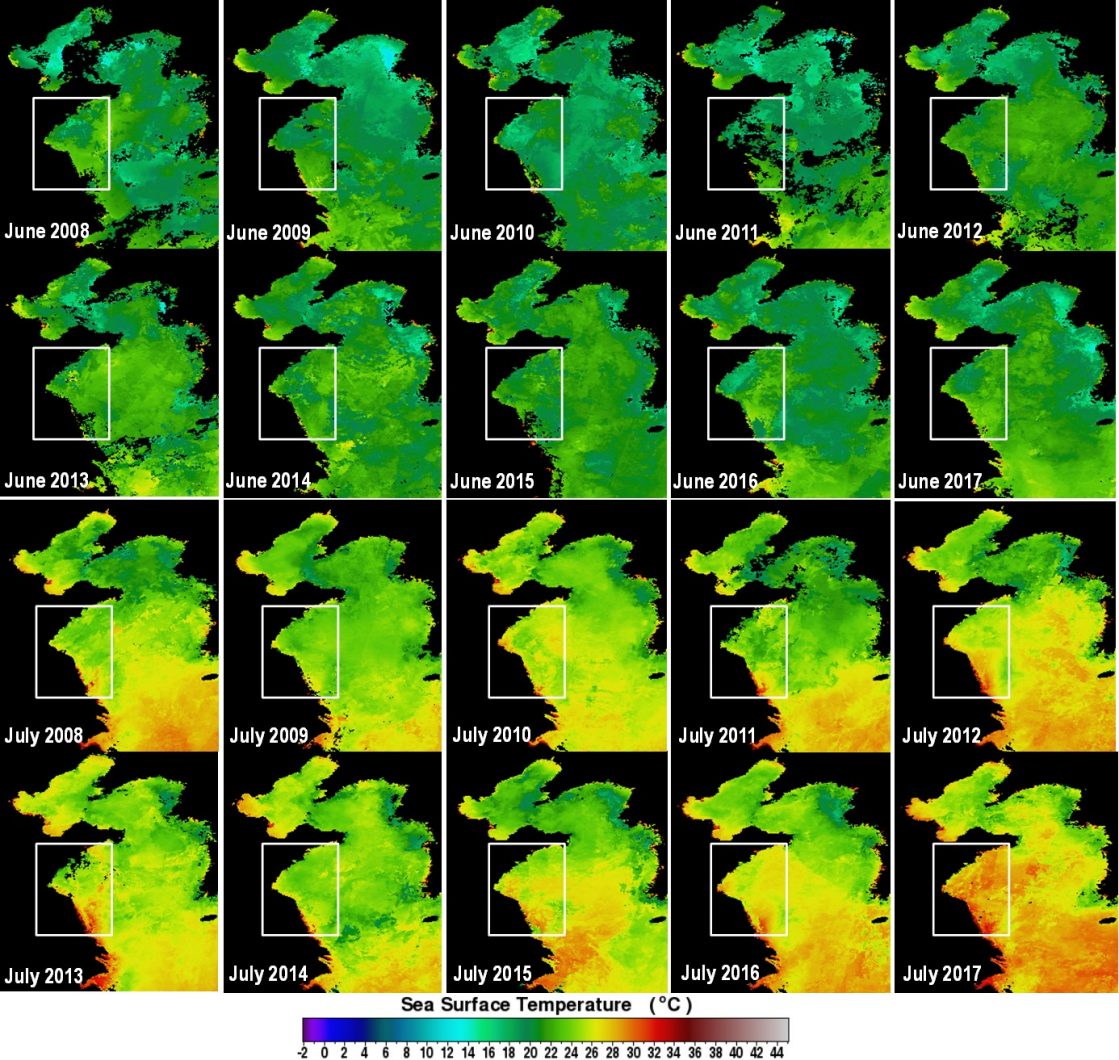
Monthly averaged Sea Surface Temperature (SST) in June and July from 2008 to 2017. (Data source: NASA data distribution system, http://oceancolor.gsfc.nasa.gov; the part shown in black color is land or clouds; white rectangles show the area of green-tide).

Sea surface temperature (SST) in the central-western Yellow Sea showed a general degree of consistency within months between years (Fig 3). Temperatures in June ranged from 18°C to 25°C, with a gradient of cooler water in the Northern Yellow Sea and warmer water along the coast of Jiangsu Province in the Southern Yellow Sea. In July from 2008 to 2016, temperatures were warmer, ranging from 24°C to 30°C (mostly 26°C to 28°C), while coastal waters were warmer than offshore and waters in the north were cooler than in the south. However, in July of 2017, temperature was much higher, ranging from 27°C to 34°C, with the water in the coastal area in which blooms occurred always being above 29°C.

### Annual variations in the *Pyropia* aquaculture area in the Yellow Sea

**Fig 4:**
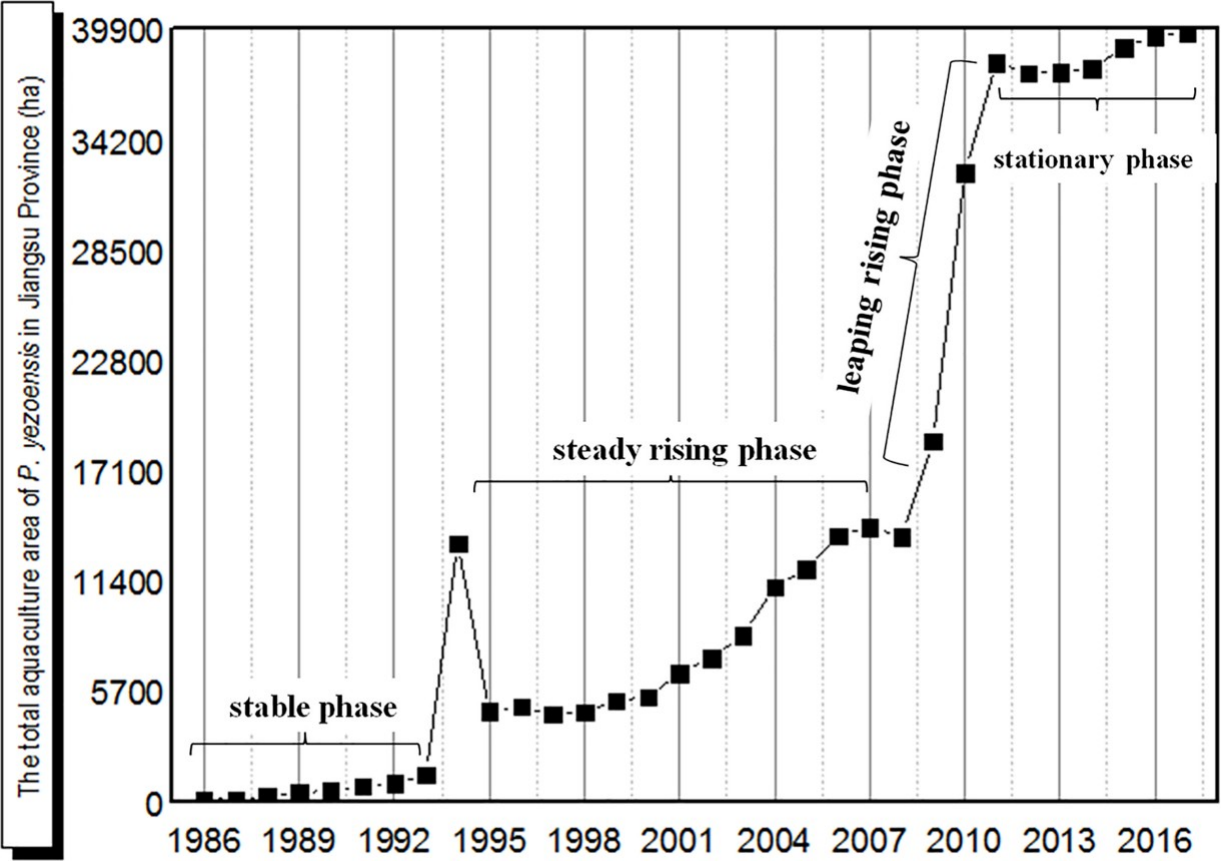
Annual variation and development process of the total aquaculture area for *P*. *yezoensis* in the Yellow Sea. (data from the China Fishery Statistical Yearbook).

Based on data from the China Fishery Statistical Yearbook (Fig 4 and S1 Table), development of *Pyropia* aquaculture since 1986 has passed through four phases. During 1986–1993, which is known as the stable phase, the total aquaculture area stayed below 1,500 ha. Afterwards, a steady increase in *Pyropia* aquaculture area occurred from 1994 to 2008, during which time the total area increased by almost three times to 14,000 ha. In the third phase, which was known as the rapid rising phase, which means, the aquaculture area rose sharply by three times to 37,674 ha from 2008 to 2012. Since 2013, the *Pyropia* aquaculture area has remained at a high level of 39,000 ha and entered a stationary phase.

**Fig 5:**
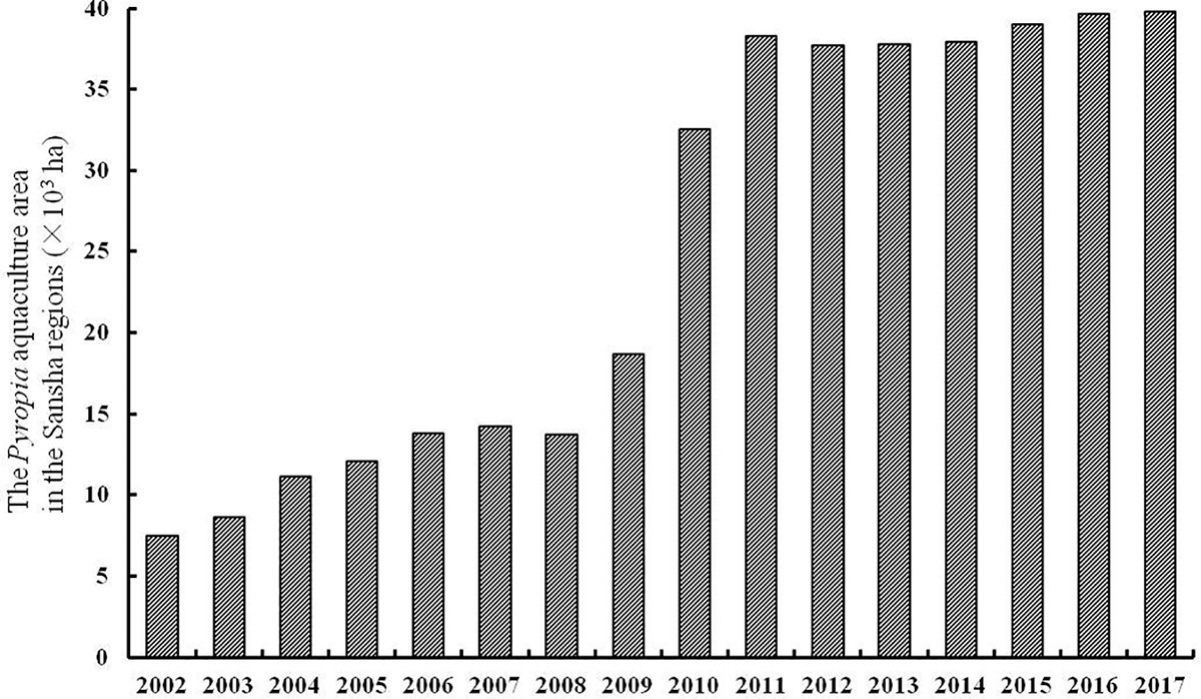
Process of *Pyropia* aquaculture area rising in the Sansha regions.

Furthermore, the *Pyropia* aquaculture area was mainly distributed in the near coastal area of the southern Yellow Sea in the early 2000s, with a few distributed in the open sea of “Sansha” regions as defined by Zhang et al. [12]. However, during 2006–2017, the percentage of *Pyropia* aquaculture area in the Sansha regions to the total area increased rapidly to 65% (Fig 5), indicating that the increased area for *Pyropia* was mainly distributed in the Sansha regions.

### Annual variation of total biomass for the attached *Ulva* macroalgae in the *Pyropia* aquaculture area

**Fig 6:**
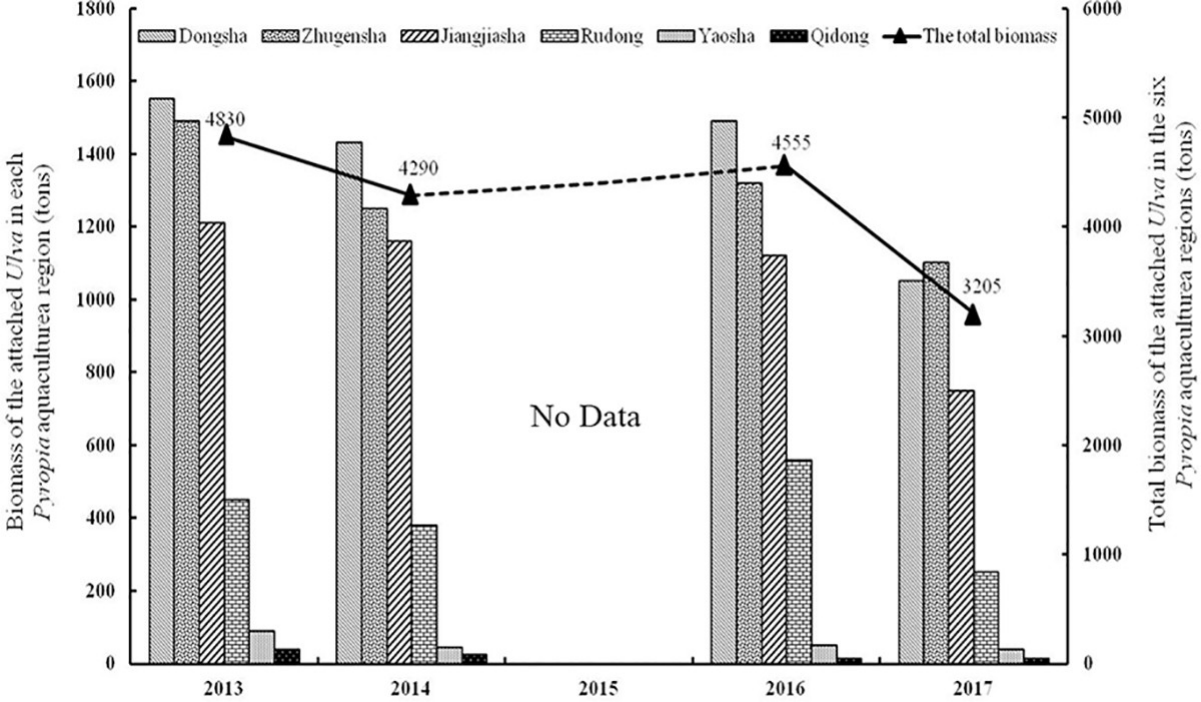
The biomass of attached *Ulva* species in *Pyropia* aquaculture area during 2013–2017 (No data in 2015).

In April from 2013 to 2017, during the early stage of the *Pyropia* harvest season, *Ulva* spp. were widely distributed on aquaculture facilities including rafts, ropes and nursery nets. From the perspective of distributed characteristics of *Pyropia* aquaculture, the attached *Ulva* biomass in the Sansha region accounted for almost 80% of the total aquaculture area. From the perspective of annual variation of attached *Ulva* biomass, the total biomass achieved 4830 tons, 4290 tons and 4555 tons in 2013, 2014 and 2016 (S2 Table), respectively. However, in 2017, the total biomass was reduced significantly by one third to 3205 tons (Fig 6 and S2 Table).

## Discussion

### Why did green tide start after 2007?

The satellite remote sensing method was used to track green tides back to 2004, and no evidence of floating *Ulva* macroalgae were observed in the Yellow Sea during 2004–2006 [10]. The first report of green tides was in summer of 2007, when the first bloom was only 191.8 km^2^ [21], which was the quite low compared with later years (Fig 2).

Previous studies have confirmed that the great amount of attached *Ulva* macroalgae in the *Pyropia* aquaculture area strongly supported the initial occurrence and formation of the blooms [7, 9, 12, 13]. During the *Pyropia* harvest season, the *Ulva* macroalgae as the fouling seaweed were cleaned and thrown away in the field artificially or mechanically at low tide. However, at high tide, the *Ulva* macroalgae with tubular morphology could easily float on the sea surface and become the source of the blooms [23]. Before 2007, most *Pyropia* aquaculture took place in the intertidal zone near the coastline. The radial sand ridges of the Jiangsu coast as a natural geographical barrier prevented the floating *Ulva* in coastal area from drifting into the Northern Yellow Sea. In addition, the reversing movement of tidal currents dominates the hydrodynamic conditions in these areas [24], making it difficult for green algal fragments to get transported to offshore waters. However, as shown in Fig 4, the *Pyropia* aquaculture area showed a rapid increase during 2007–2009, with the rapid expansion occurring in the Sansha regions on the sand shoals where is almost 40 km away from the coastline and is much closer to Northern Yellow Sea. With the relative shorten distance and without the geographical barrier, combined with dominant south-east wind-driven currents and resultant upwelling between the Jiangsu coast and the western Yellow Sea, the attached *Ulva* which were dragged down into the sea could quickly drift offshore into the Northern Yellow Sea in the next two or three months.

### Why did green tide decrease significantly in 2017?

#### Rise of golden tides in the Yellow Sea

**Fig 7:**
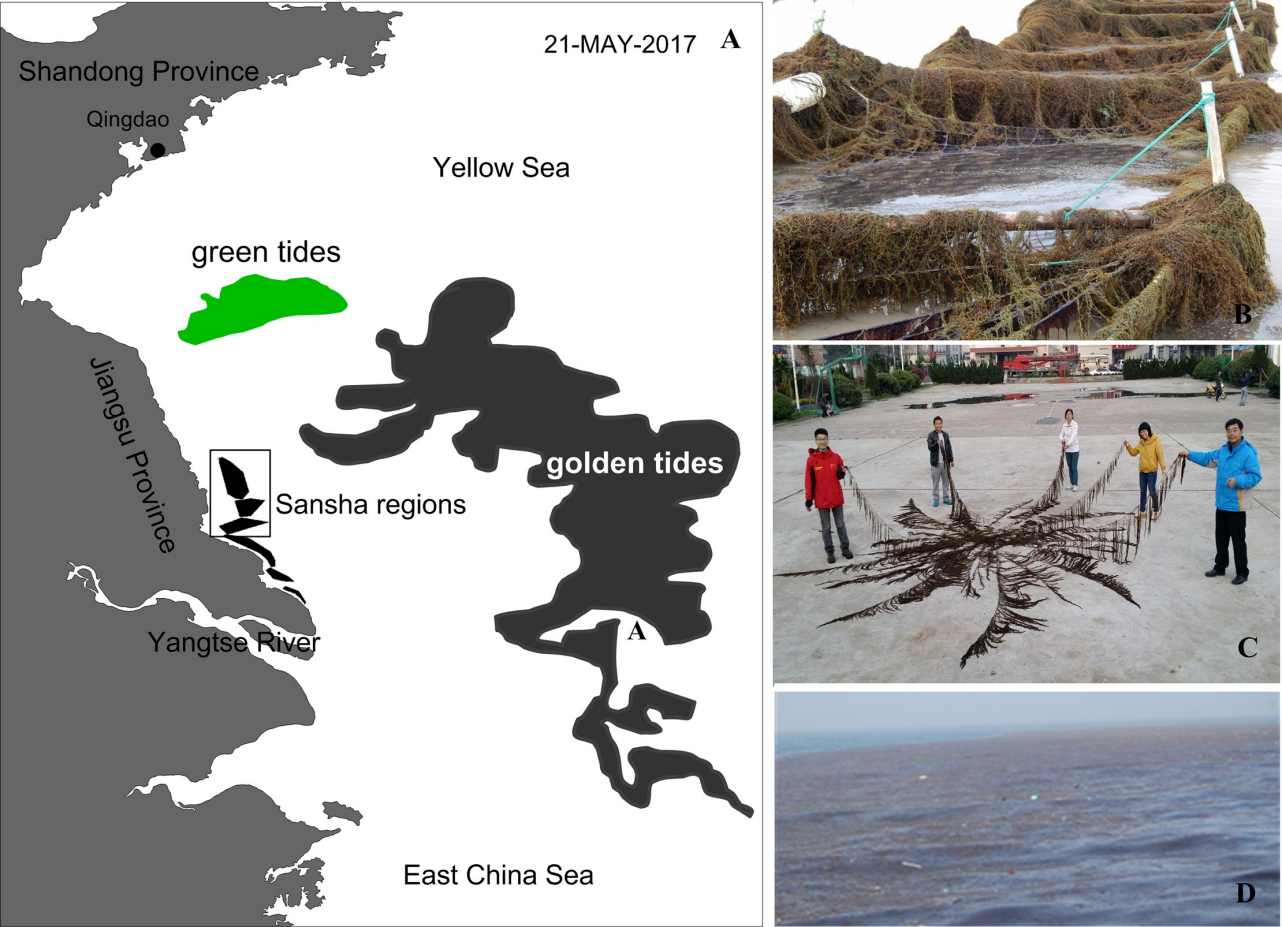
The distribution of green tides and golden tides in the Yellow Sea and East China Sea. (A) The distribution of green tides and golden tides in the Yellow Sea and East China Sea on May 21, 2017. (B) The *Pyropia* aquaculture was completely covered by floating *Sargassum* seaweed in the Yellow Sea. (C) An individual floating *Sargassum* seaweed collected from the East China Sea. (D) Free-floating golden tides in the Yellow Sea.

In the Yellow Sea of China, the occurrence of macroalgal blooms known as golden tides caused by *Sargassum* seaweed has been increasing rapidly [25–27]. The *Sargassum* thallus is leathery, tough and differentiated into features that resemble leaves and a stem and has well-developed gas bladders for flotation. Moreover, individual *Sargassum* seaweeds can reach up to 4 m long (Fig 7C). In previous years, golden tides occurred without drawing attention because they drifted to the deep sea and caused no damage to coastal areas. However, in 2017, unusual currents and winds drove floating *Sargassum* thalli with a distributed area of 47,713 km^2^ to the coast of the Yellow Sea (Fig 7A and 7D), resulting in dramatic damage to *Pyropia* aquaculture (Fig 7B). (The satellite date source of Fig 7A: North China Sea Brach of SOA monitor date distribution system for green tides, http://www.ncsb.gov.cn/n1/n127/n139/n39/n48) (The individual in this manuscript has given written informed consent (as outlined in PLOS consent form) to publish these case details).

**Fig 8:**
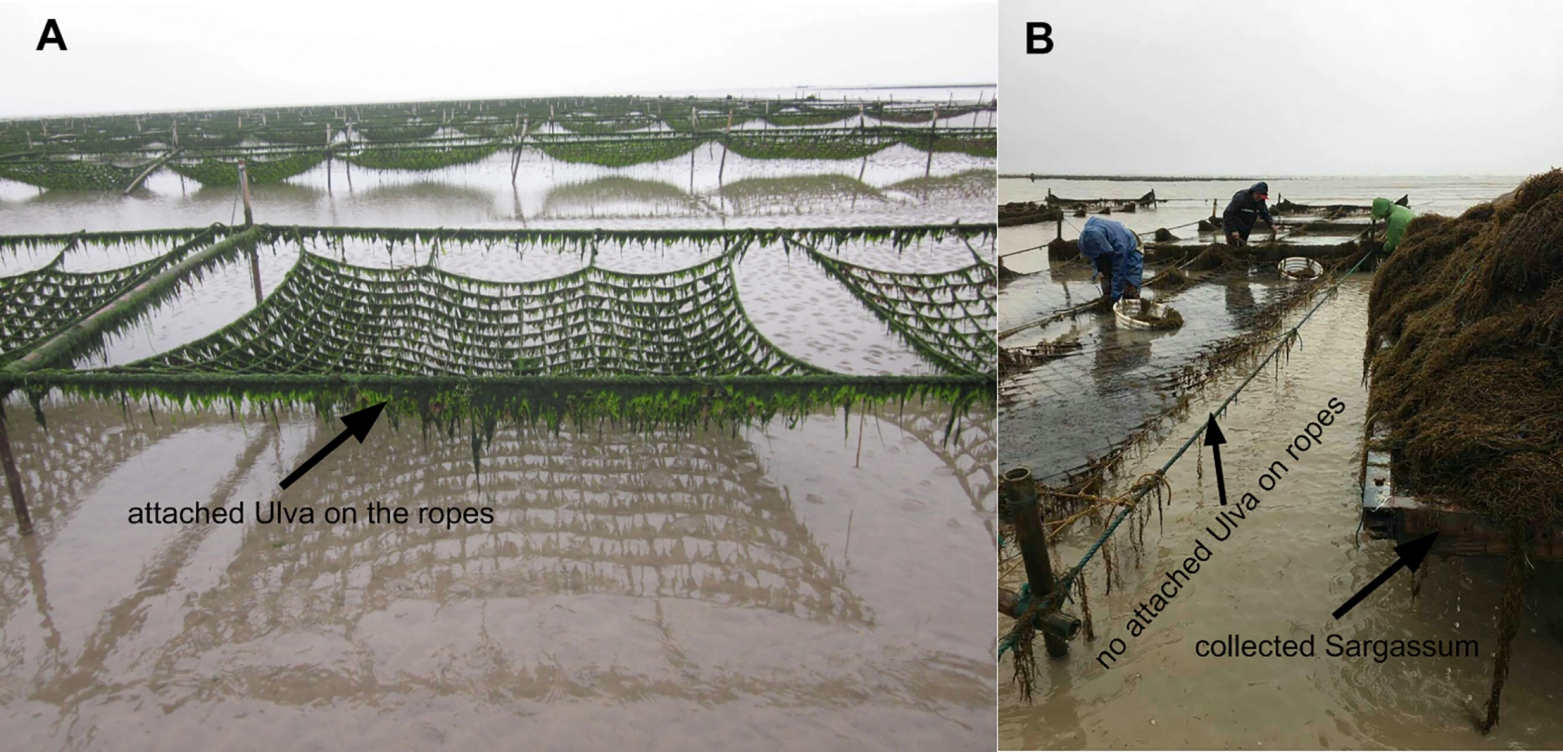
Variation in biomass of attached *Ulva* on the *Pyropia* aquaculture ropes after golden tides occurred. (A) A large amount of *Ulva* biomass attached on the *Pyropia* aquaculture ropes. (B) The *Sargassum* were harvested from the *Pyropia* aquaculture areas and no *Ulva* species were observed on the ropes.

In recent years, eutrophication has been increasing in the Yellow Sea, and a common symptom of eutrophication is profuse blooms of marine macroalgae, which are formed by the excessive growth of some macroalgal species living in the intertidal zone. Indeed, macroalgal blooms are now widespread in coastal areas worldwide. On May 21^st^, 2017, the golden tide became much larger than the green tide in the Yellow Sea, possibly as a result of competition for nutrients. Another reason may have been that the floating golden tide attacked the *Pyropia* aquaculture area, resulting in its occupying the space previously utilized by *Ulva*, resulting in the loss of one third of the attached *Ulva* macroalgae in 2017 compared with previous years (Fig 6). The ropes, as one of the components of *Pyropia* aquaculture infrastructures, are the most important attached substrate for *Ulva* species, and the attached *Ulva* on ropes provide almost 2,500 tons of initial biomass for blooms each year [12]. However, in 2017, we did not observe the attached *Ulva* on the ropes in areas in which the golden tides occurred (field observation, Fig 8), and this lost biomass further reduced the biomass floating *Ulva* blooms.

As I mentioned previously, eutrophication is severe in coastal area in the Yellow Sea of China. Official monitoring of nutrients in the Yellow Sea by the State Oceanic Administration of China indicated that nutrients in 2017 kept a high level, and were almost same with previous years (Date source: http://www.soa.gov.cn/zwgk/hygb/zghyhjzlgb/, Annual Bulletin of China Marine Ecological Environment Status). Consequently, the variation of nutrients could not be the reason causing the reduction of the blooms in 2017.

#### Extreme high temperature inhibited the rapid growth of *U*. *prolifera*

Growth rates of green tide algae could be regulated by environmental parameters [28, 29]. Previous studies of eight marine algal species associated with green-tides revealed that temperature played a vital role in controlling their growth [5, 30]. In recent years, during June to July, sea surface temperature (SST) of the Yellow Sea ranged from 20 to 27°C [10], which is the optimal temperature for the growth of *U*. *prolifera* [31]. However, in 2017, extreme high temperature (> 30°C, Fig 3) lasted for more than 1 month, resulting in the slow expansion of the blooms.

Irradiance also had a strong effect on spore release, germination and the growth of macroalgae [32]; thus, light was also an important factor in the recruitment or decline of green tide algae. *U*. *prolifera* can tolerate high levels of irradiance, and is able to grow under levels as high as 600 μmol m^-2^ s^-1^ [31]. The surface irradiance of the entire Yellow Sea can be maintained at over 1,000 μmol m^-2^ s^-1^ which was difficult to be realized under laboratory conditions [33]. Generally, high irradiance will also occur when the temperature is quite high during summer. In previous years, the shading effect of suspended solids and upper algae in the Southern Yellow Sea resulted in light playing an important role in the growth of green algae. However, we inferred that the extreme high irradiance also became the factor inhibiting the fast growth of *Ulva* macroalgae in 2017.

#### Application of controlling attached and floating *Ulva* in the original sea area

*Ulva prolifera*, the dominant species of the blooms, is an edible macroalga characterized by high nutritional value that can be used as fertilizer, fodder and even human food. In the Southern Yellow Sea, floating *Ulva* seaweed was bright green and the protein content can reach up to 30% [34], making it a good material for food. An *Ulva* food processing machine was made in Jiangsu Province in conjunction with our research team. Based on an average growth rate of ~30% for *U*. *prolifera* [12], if one ton of *Ulva* in the original sea area can be harvested and processed to food, 2,700 tons of floating blooms can be removed from the Qingdao coast of the Northern Yellow Sea. In 2017, the local oceanic administration organized fishermen to harvest approximate 300 tons of floating *Ulva* species (personal communication), which accounted for almost 1/10 of the total biomass of the attached *Ulva* in the *Pyropia* aquaculture area. Accordingly, this likely contributed to the reduction of *Ulva* blooms in 2017.

In addition, the high economic benefits of *Ulva* products have been widely publicized; therefore, some fishermen have begun to collect attached or floating *Ulva* seaweed and sell it for further income. However, most fishermen have not yet begun to do this. In Zhejiang Province, a seaweed company conducted *Ulva* aquaculture, produced many *Ulva* products and exported them to other countries. We believe that the *Ulva* seaweed processing industry will become an emerging market and that it has the potential to provide many jobs while controlling *Ulva* blooms.

According to monitoring information from the Environmental Monitoring Center of the East China Sea (unpublished), golden tides also occurred in the Yellow Sea in 2012; however, we do not have detailed data on the attached *Ulva* biomass in the *Pyropia* aquaculture areas in 2012. Therefore, competition between *U*. *prolifera* and *Sargassum* sp., nutrient variation and consecutive environmental monitoring should be conducted to better understand the annual variations in *Ulva* blooms in the Yellow Sea.

## Supporting information

S1 Table Data of annual variation and development process of the total aquaculture area for *P*. *yezoensis* in the Yellow Sea. (PDF)

S2 Table Data of The biomass of attached *Ulva* species in *Pyropia* aquaculture area during 2013–2017 (No data in 2015). (PDF)
