# Supplementary material for: Annual patterns of macroalgal blooms in the Yellow Sea during 2007–2017
Source: PLoS One. 2019 Jan 14;14(1):e0210460. doi: 10.1371/journal.pone.0210460 (PMC6331115; doi:10.1371/journal.pone.0210460)
Supplement: S1 Table — (PDF) [file pone.0210460.s001.pdf]

Year	Total aquaculture area for *P. yezoensis*
1985	
1986	190
1987	190
1988	290
1989	490
1990	640
1991	830
1992	990
1993	1430
1994	13410
1995	4700
1996	4950
1997	4560
1998	4670
1999	5250
2000	5480
2001	6680
2002	7470
2003	8650
2004	11140
2005	12070
2006	13800
2007	14250
2008	13730
2009	18670
2010	32560
2011	38260
2012	37674
2013	37765
2014	37943
2015	39024
2016	39618
2017	39758
