# Supplementary material for: Annual patterns of macroalgal blooms in the Yellow Sea during 2007–2017
Source: PLoS One. 2019 Jan 14;14(1):e0210460. doi: 10.1371/journal.pone.0210460 (PMC6331115; doi:10.1371/journal.pone.0210460)
Supplement: S2 Table — (PDF) [file pone.0210460.s002.pdf]

Year	Total	Dongsha	Zhugensha	Jiangjiasha	Rudong	Yaosha	Qidong
2013	4830	1550	1490	1210	450	90	40
2014	4290	1430	1250	1160	380	45	25
2015	-	-	-	-	-	-	-
2016	4555	1490	1320	1120	560	50	15
2017	3205	1050	1100	750	250	40	15
